# Annotations

**Published:** 1909-04-10

**Authors:** 


### ANNOTATIONS.

### The Social Problems of Tuberculosis.

At the time of the late International Tuberculosis Congress at Washington some uncertainty attended the reports of the actual and authoritative resolutions passed by that body at the termination of the meeting. The version of the official delegates of the United Kingdom (Dr. Newsholme for England and Messrs. MacDougall and Stafford for Scotland and Ireland respectively) is now available, and reads as a temperate, well-considered pronouncement upon the social problems involved in the disease in question, although perhaps very few now living are likely to see established "night-camps for ambulant cases of tuberculosis which cannot enter hospitals and sanatoria." The conveyance of infection from man to man is designated the most important source of the disease, the corollaries of segregation of advanced cases and obligatory notification receiving due mention. Unfortunately the discussion of these measures, although they are in all probability highly necessary, is only too likely to inflame inconveniently that dread of infection which the lay mind so readily entertains. More hospitals and sanatoria are called for, and legislation for the hygienic regulation of factories and dwelling-houses approved. All this, down to the provision of children's playgrounds, means money; in fact, these resolutions do but reiterate the truth that all anti-tuberculosis measures are largely a question of economics. Perhaps the really most profitable field for medical precept lies in the instruction in personal hygiene which is recommended in all schools for the professional training of teachers. In the case of the British lower classes—and, of course, they form the bulk of the nation—the taste for the open window and the clean epidermis must be inculcated early, or not at all.

### A Scale of Fees.

An interesting attempt to set up a standard scale of fees in the capital of South Australia, Adelaide, has been recently made by the local branch of the British Medical Association. It is to be understood that the scale binds no one, not even the members of the Association; but it has been drawn up and recommended, and as the charges are the minimal, it can only be said that it is to be wished that the same held good in Britain. An ordinary visit "first class" is from half a guinea upwards, and "second class" from seven and six. We are not told how the distinction is made; but in a country where the poorest are less poor and the wealthiest less wealthy than here, it is probably easier to divide patients into two classes only than in Great Britain. An evening visit is labelled seven and six to one guinea; a night visit (11P.M. to 8 A.M.) one guinea and upwards. Mileage by day is reckoned at five shillings, by night ten shillings, per mile. When a visit is paid to more than one member of a family, and the fees are paid by one person, half fees are to be charged for each person after the first. Major operations, however, seem to be cheap in Australia: from ten guineas upwards is laid down, exclusive of after attendance. Minor operations are scheduled at two to ten guineas, and administration of a general anæsthetic are one guinea and upwards. An interesting generalisation is that in which it is held that in the case of major operations the anæsthetist and the assistant should each receive a sum equal to 10 per cent. of the operator's fee. This sum total seems fair, but in view of the much greater importance and responsibility of the anæsthetist's position, we should prefer to see the percentages revised in his favour to twelve and a half and seven and a half respectively. There are also a number of scales for contract practice given, and for expert evidence, insurance work, certificates of lunacy, and so forth. Of these it need only be said that they do not differ much from fees in this country, except for club practice; in respect of the latter the South Australian schedule is one to make the mouth water of any club doctor in this country.

### Compensation Problems.

To the knotty points, both legal and medical, arising out of the various Workmen's Compensation Acts of recent years, reference has more than once been made in these pages; and the supply shows no signs of diminution. Thus a remarkable case came before the Appeal Court on March 26, concerning which the decision of the County Court was reversed. The circumstances were, briefly, that a workman sustained a mutilating wound of the right hand so severe that he barely escaped amputation. However, by careful treatment, including operative manipulations under chloroform, the hand was saved, and he was left with a member which would in time have been functionally nearly perfect, provided contraction were avoided. In order to secure this he was again anæsthetised for the purpose of skin grafting, but unfortunately died under chloroform. The Court of Appeal found that the second operation was in reality a postponed but essential part of the first one, and performed with the object of restoring the man to working life in the interests of those from whom compensation was due; and thereon, that the case must be treated on the same footing as if the original accident had caused death. We quote this case as a sample merely because it is quite recent; scores of similar difficult problems have come into Court for decision, and those who see much of this work, whether from the legal or the medical aspect, are reaching conclusions which show remarkable unanimity. There seems no doubt that malingering has been directly fostered by these Acts. Two points connected therewith touch especially our profession. One is the disgraceful arrangement which has sprung up, according to an eminent lawyer, between a certain sort of practitioner and an even more despicable kind of solicitor, to the effect that the former, when giving evidence in support of a claim, is to receive fees only if the suit is successful. The other is the pressing need for the presence of competent medical assessors to assist County Court judges in those not infrequent cases where a conflict of opinion arises, honestly or otherwise, between the medical witnesses produced by the two sides.

